# Mechanisms of ATII-to-ATI Cell Differentiation during Lung Regeneration

**DOI:** 10.3390/ijms21093188

**Published:** 2020-04-30

**Authors:** Mohit Aspal, Rachel L. Zemans

**Affiliations:** 1 College of Literature, Science and the Arts, University of Michigan, Ann Arbor, MI 48109, USA; aspalmoh@umich.edu; 2 Division of Pulmonary and Critical Care Medicine, Department of Internal Medicine, University of Michigan, 109 Zina Pitcher Place, Ann Arbor, MI 48109-2200, USA; 3 Program in Cellular and Molecular Biology, University of Michigan, Ann Arbor, MI 48109, USA

**Keywords:** alveolar epithelium, lung injury, lung regeneration

## Abstract

The alveolar epithelium consists of (ATI) and type II (ATII) cells. ATI cells cover the majority of the alveolar surface due to their thin, elongated shape and are largely responsible for barrier function and gas exchange. During lung injury, ATI cells are susceptible to injury, including cell death. Under some circumstances, ATII cells also die. To regenerate lost epithelial cells, ATII cells serve as progenitor cells. They proliferate to create new ATII cells and then differentiate into ATI cells. Regeneration of ATI cells is critical to restore normal barrier and gas exchange function. Although the signaling pathways by which ATII cells proliferate have been explored, the mechanisms of ATII-to-ATI cell differentiation have not been well studied until recently. New studies have uncovered signaling pathways that mediate ATII-to-ATI differentiation. Bone morphogenetic protein (BMP) signaling inhibits ATII proliferation and promotes differentiation. Wnt/β-catenin and ETS variant transcription factor 5 (Etv5) signaling promote proliferation and inhibit differentiation. Delta-like 1 homolog (Dlk1) leads to a precisely timed inhibition of Notch signaling in later stages of alveolar repair, activating differentiation. Yes-associated protein/Transcriptional coactivator with PDZ-binding motif (YAP/TAZ) signaling appears to promote both proliferation and differentiation. We recently identified a novel transitional cell state through which ATII cells pass as they differentiate into ATI cells, and this has been validated by others in various models of lung injury. This intermediate cell state is characterized by the activation of Transforming growth factor beta (TGFβ) and other pathways, and some evidence suggests that TGFβ signaling induces and maintains this state. While the abovementioned signaling pathways have all been shown to be involved in ATII-to-ATI cell differentiation during lung regeneration, there is much that remains to be understood. The up- and down-stream signaling events by which these pathways are activated and by which they induce ATI cell differentiation are unknown. In addition, it is still unknown how the various mechanistic steps from each pathway interact with one another to control differentiation. Based on these recent studies that identified major signaling pathways driving ATII-to-ATI differentiation during alveolar regeneration, additional studies can be devised to understand the interaction between these pathways as they work in a coordinated manner to regulate differentiation. Moreover, the knowledge from these studies may eventually be used to develop new clinical treatments that accelerate epithelial cell regeneration in individuals with excessive lung damage, such as patients with the Acute Respiratory Distress Syndrome (ARDS), pulmonary fibrosis, and emphysema.

## 1. Introduction

There are two types of cells in the alveolar epithelium: large, flattened alveolar type I (ATI) cells that cover 95%–98% of the alveolar surface and permit gas exchange and cuboidal alveolar type II (ATII) cells that are the progenitor cells responsible for regenerating ATI and ATII cells during homeostasis and after injury [1,2,3]. The lung epithelium serves as a barrier that protects the body from airborne pathogens and prevents leakage of bodily fluids into the airspaces. In the event of epithelial cell death, such as during infection or after exposure to cigarette smoke, the barrier is compromised. During homeostasis, alveolar epithelial cells have a long but limited lifespan [1]. After the death of an occasional alveolar epithelial cell, barrier function is maintained by adequate levels of ATII cell proliferation and differentiation to replace the lost cells. During lung injury, excessive epithelial cell death results in impaired barrier function. ATI cells are particularly susceptible to injury, but ATII cells can die in cases of severe or certain types of injury. 

It has long been known that ATII cells are the principal progenitor responsible for regenerating the injured alveolar epithelium [1,4,5,6], although it is increasingly recognized that other progenitors can be mobilized under certain circumstances [7,8,9]. ATII cells proliferate to replace lost cells, and once sufficient cell numbers have been restored, some differentiate into ATI cells to restore normal alveolar structure. Since ATI cells are largely responsible for barrier function and gas exchange, the regeneration of ATI cells is absolutely critical to restore normal lung function. Signaling pathways that promote ATII cell proliferation have been identified by us and others and include keratinocyte growth factor (KGF), hepatocyte growth factor (HGF), epidermal growth factor (EGF), Wnt/β-catenin, forkhead box protein M1 (FoxM1), and others [10,11,12,13,14,15,16,17,18,19,20]. Although we have some understanding of the mechanisms that drive ATII cell proliferation, the signaling pathways that drive ATII-to-ATI cell differentiation have remained elusive. Recently, in large part due to the emergence of lineage tracing technology [21], several studies have uncovered various signaling pathways shown to be involved in this differentiation step: Wnt/β-catenin, Notch, YAP/TAZ, BMP, and TGFβ. Here, we discuss this recent work and the contribution these studies have made to advance our understanding of lung regeneration. We also briefly discuss future research questions that can be examined based on the strong foundation established by this handful of studies. For example, the various signaling pathways identified to regulate ATI cell differentiation undoubtedly interact with one another in a coordinated manner to control differentiation. However, the way they interact—whether they operate upstream, downstream, or parallel to one another—is still unknown. In addition, future studies will be necessary to confirm the extent to which these pathways, identified in mouse models of regeneration, are active in the human lung. Strengthened understanding of the mechanisms involved in ATII-to-ATI cell differentiation ultimately may lead to development of new clinical treatments that accelerate lung repair in individuals with excessive lung damage, such as patients with Acute Respiratory Distress Syndrome (ARDS), pulmonary fibrosis, and emphysema. 

## 2. Wnt/β-Catenin Signaling

We previously reported that inhibition of Wnt/β-catenin signaling prevented ATII cell proliferation during regeneration after lung injury [13]. More recently, elegant studies using lineage tracing and inducible ATII cell-specific gene deficient mice have confirmed that Wnt/β-catenin signaling is critical for ATII cell proliferation after lung injury in multiple models [14,15]. Interestingly, these studies also identified a small subset of ATII cells that function as alveolar stem cells during homeostasis. These stem cells are responsible for maintaining ATII cells during homeostatic turnover and do so in a Wnt/β-catenin-dependent manner. Moreover, during homeostasis, adjacent PDGFRα+ fibroblasts are the source of secreted Wnts, which maintain the ATII cells and are thus considered to be the niche of the ATII stem cells. Wnt signaling is required not only for ATII cell proliferation but also for maintenance of the ATII cell phenotype, as knockout of β-catenin induces differentiation into ATI cells. Following lung injury, the larger population of ATII cells that is mobilized to proliferate themselves produce Wnts, stimulating proliferation via autocrine signaling even in ATII cells outside the niche [14]. Presumably, the Wnt signaling that maintains the ATII cell phenotype during homeostasis also does so during ATII cell proliferation during regeneration and must be downregulated to permit ATII-to-ATI differentiation. 

## 3. Notch Signaling

Notch signaling has been strongly implicated in proliferation and differentiation in many organs. After embryonic tissue development, such as the development of the lung epithelium, the expression of the Notch ligand delta-like 1 homolog (Dlk1) disappears, returning only in some adult tissues undergoing regeneration. A recent landmark study uncovered the role of Notch, specifically Dlk1, in alveolar regeneration. In acute lung injury, temporal regulation of Notch signaling by Dlk1 was shown to have a role in alveolar repair, promoting ATII-to-ATI differentiation. In *Pseudomonas aeruginosa*-induced mice lung injury model, Dlk1 leads to a precisely timed inhibition of Notch signaling in later stages of alveolar repair, which activates differentiation [22]. The regenerative role of Dlk1 in alveolar differentiation was supported by several experimental studies. Experiments using inducible ATII cell-specific *Dlk1* mutant mice, lineage-tracing studies, RNA-seq, Notch reporter and ATII-specific constitutively active Notch mice revealed that Notch signaling is initially activated in ATII cells during the proliferation phase, but that later, Notch signaling is downregulated by Dlk1 as ATII cells differentiate into ATI cells [22]. This high-to-low Notch switch was essential for ATII cell differentiation into ATI cells. In ATII cell-specific *Dlk1* conditional knockout mice, high Notch activation is sustained. This results in delayed ATI cell differentiation and the accumulation of an intermediate cell population of alveolar epithelial cells that expressed low levels of both ATI and ATII cell markers. This phenotype was partially rescued by Notch inhibition [22]. In conclusion, Notch signaling is activated during the proliferation phase of alveolar regeneration but is later deactivated due to Dlk1 upregulation, promoting ATII-to-ATI cell differentiation. However, a key remaining unknown is how Dlk1 expression is regulated. If Dlk1 upregulation is a critical signal for inducing ATI cell differentiation, understanding the factors upstream of Dlk1 expression will be key for understanding the overall regulation of ATII-to-ATI cell differentiation. 

## 4. BMP/SMAD Signaling

Bone morphogenetic protein (BMP) signaling in mammalian systems has been shown to play a variety of complex roles in proliferation and differentiation in many organs. Recently, a seminal study demonstrated that dynamic changes in BMP signaling play a critical role in alveolar regeneration [23]. BMP signaling is active in the vast majority of ATII and ATI cells during homeostasis. During regeneration, BMP signaling is downregulated during ATII cell proliferation and then upregulated during ATI cell differentiation. This activation and deactivation of BMP signaling is attributable to dynamic expression of BMP ligands, receptors, and antagonists. Moreover, using both pharmacologic and genetic approaches in cultured alveolar organoids and mice, the investigators demonstrated that BMP inhibits ATII cell proliferation and promotes ATII-to-ATI cell differentiation. Interestingly, the fibroblasts that constitute the ATII cell niche also display a reduction in BMP signaling during ATII cell proliferation, with a rebound during ATII-to-ATI cell differentiation. BMP gain of function in the fibroblasts had no effect on fibroblast proliferation but similarly inhibited ATII cell proliferation [23]. Taken together, these data suggest that during homeostasis, active BMP signaling maintains ATII cell quiescence; during regeneration, deactivation of BMP signaling promotes ATII cell proliferation, whereas reactivation of BMP signaling promotes ATI cell differentiation. This finding establishes a strong foundation upon which future questions may be addressed: What is the mechanism by which BMP signaling inhibits ATII cell proliferation? Does it directly inhibit the cell cycle or does it prime ATII cells to be less responsive to known mitogens such as KGF, HGF, Wnt, and EGF? Similarly, how does BMP signaling induce ATI cell differentiation? More generally, the observations that BMP signaling simultaneously inhibits proliferation and drives differentiation suggests that proliferation may not be a necessary prerequisite for differentiation. Finally, the mechanisms by which BMP signaling in fibroblasts limits their ability to promote ATII cell proliferation are unknown at this time. 

## 5. Yap/Taz Signaling

YAP and TAZ are important transcription coactivators in the Hippo signaling pathway known to be involved in embryonic development, homeostasis, and tissue regeneration after injury. An elegant recent study has shown YAP/TAZ to play a crucial role in ATII-to-ATI differentiation in alveolar repair [24]. In the Streptococcus pneumoniae model of lung injury, YAP and TAZ expression and nuclear localization increased in ATII cells after lung injury. Moreover, ATII cell specific Yap/Taz knockout mice displayed impaired ATII cell proliferation and ATII-to-ATI cell differentiation during regeneration. These mice also developed fibrotic lesions, consistent with the widely accepted view that impaired epithelial regeneration begets fibrosis. The expression of several genes known to promote cell proliferation and differentiation, including FGFs, Wnts, EGFR, and BMP4, were reduced in YAP/TAZ deficient ATII cells, although it is as of yet unknown whether these genes mediate the role of YAP/TAZ signaling in ATII cell proliferation and differentiation. Another study used alveolar organoid cultures and the bleomycin model of lung injury to demonstrate the role of TAZ signaling in ATII-to-ATI cell differentiation [25]. In this study, TAZ nuclear localization was observed in ATI but not ATII cells. Moreover, pharmacologic inhibitors and TAZ knockout prevented ATII-to-ATI cell differentiation in organoids. In the bleomycin lung injury model, conditional deletion of TAZ in ATII cells also led to reduced ATI cell regeneration and, as in the S. pneumoniae model, resulted in greater fibrosis. Interestingly, the ATII cell-specific YAP/TAZ knockout mice exhibited prolonged inflammation, apparently due to an inability to upregulate Inhibitor-of-NFκB, alpha (IκBα), a repressor of Nuclear-Factor κB (NFκB). Chromatin immunoprecipitation (ChIP) experiments in an alveolar epithelial cell line suggested that IκBα is a direct YAP/TAZ target gene. Moreover, Yap/Taz knockdown inhibited, whereas Yap/TAZ overexpression increased, IκBα. Moreover, IκBα overexpression attenuated the prolonged inflammation, delay in alveolar regeneration, and fibrosis observed in YAP/TAZ knockout mice. In contrast to the S. pneumoniae model, there was no defect in proliferation of TAZ KO ATII cells. A third study using the pneumonectomy model of compensatory lung growth revealed that YAP signaling is activated in ATII cells and ATII cell specific deletion of YAP inhibited ATII cell proliferation and ATII-to-ATI cell differentiation [26]. YAP inhibition also decreases proliferation during homeostasis [27]. However, despite its role in ATII-to-ATI cell differentiation during regeneration [24,25,26] and development [25], it appears that this pathway is dispensable for normal ATI cell turnover during lung homeostasis.

## 6. Recruited Macrophages

During alveolar regeneration in many models, the lung monocyte/macrophage population expands. Monocyte-derived macrophages are recruited from the circulation, and resident alveolar macrophages proliferate [28,29,30]. Some of these macrophages contribute to epithelial injury [31], but some are likely to contribute to repair. A landmark study showed that preventing the recruitment of monocyte-derived macrophages via genetic deletion of the monocyte chemokine receptor CCR2 impaired ATII cell proliferation and ATII-to-ATI cell differentiation [32]. The recruited macrophages displayed an M2-like gene expression signature, consistent with their reparative phenotype. Knockout of the receptor that skews macrophages towards an M2 phenotype, IL4RA, resulted in impaired regeneration. Additional experimentation suggested that IL13 derived from ILC2s may contribute to M2 polarization during lung regeneration. Taken together, these data suggest a critical role for recruited M2 macrophages in alveolar regeneration. Although the full panel of mediators that macrophages secrete to induce alveolar regeneration remains to be determined, a couple of studies have elucidated a role for macrophage-derived mediators in ATII cell proliferation. One elegant study several years ago suggested that macrophage-derived TNFα stimulates ATII cell production of Granulocyte-macrophage colony-stimulating factor (GM-CSF), which induces ATII cell proliferation via autocrine signaling [33]. A more recent study suggested that macrophage TFF2 signaling induced expression of Wnts, which promote ATII cell proliferation [34]. 

## 7. Cdc42

In a series of studies, Nan Tang et al. demonstrated that mechanical tension induced by pneumonectomy results in actin polymerization and spreading of regenerating ATII cells [26]. Under these circumstances, ATII cell-specific deletion of Cell division control protein 42 homolog (Cdc42) inhibited ATII cell proliferation and actin polymerization [26,35]. Moreover, Cdc42 deficient ATII cells fail to differentiate into ATI cells [35]. In fact, the investigators observed that new alveoli failed to form after pneumonectomy, resulting in an enlargement of existing alveoli. Eventually, these mice developed fibrosis, linking a failure of ATI cell regeneration to fibrosis. 

## 8. Etv5 Signaling

The ETS family transcription factor ETV5, previously implicated in lung development [36], was recently shown to be necessary for ATII cell proliferation and the maintenance of ATII cell identity in the adult lung. ETV5 deficiency induced ATII-to-ATI cell differentiation both in vitro and during homeostasis in vivo [37]. In the bleomycin model of lung injury, Etv5 deficiency resulted in impaired ATII cell proliferation with enhanced ATII-to-ATI cell differentiation. Etv5 deficiency also reduced ATII cell proliferation in the Kras model of lung cancer. ChIP-seq identified Etv5 binding sites in the promoters of ATI and ATII cell genes but the specific genes that were enriched and whether the regulation of gene expression is direct is unknown. 

## 9. TGFβ

To identify additional candidate signaling pathways that may regulate ATII-to-ATI cell differentiation, we performed single cell RNA sequencing on lineage tagged ATII cells during regeneration in the LPS model of lung injury [38]. We identified three transitional cell states: (1) proliferating ATII cells indicated by high expression of cell cycle markers such as *mKi67* and *Pcna*, (2) an intermediate cell state characterized by high expression of markers of cell cycle arrest such as p15 and p53 as well as downregulation of ATII cell markers and modest upregulation of ATI cell markers, and (3) differentiating ATII cells characterized by further upregulation of ATI cell markers than approaches that of mature ATI cells. Other groups subsequently identified a similar intermediate cell state in various models of regeneration [35,39,40,41], strongly suggesting that the mechanisms of regeneration are conserved regardless of the type of initial injury. Further interrogation of the gene expression profiles of these transitional cell states, including pathway analysis, revealed activation of specific pathways. Specifically, TGFβ signaling was low in the proliferating cells, highly upregulated in the intermediate cells state, and then downregulated in differentiating cells. Additional in vitro experiments suggested that TGFβ signaling is necessary to induce proliferating cells to exit the cell cycle but subsequent downregulation of TGFβ may promote differentiation. However, the role of TGFβ in alveolar regeneration in vivo and the function of the many other pathways identified by the scRNAseq studies requires additional investigation. The pathogenesis of pulmonary fibrosis is widely believed to arise from ineffectual regeneration of the alveolar epithelium after injury, although the specific regenerative defect has been unknown. Since pulmonary fibrosis is characterized by hyperplasia of alveolar epithelial cells with a morphology that is transitional between ATII and ATI cells and is driven by unchecked TGFβ activation, we hypothesized that the specific regenerative defect driving fibrosis may be an arrest in the ATII-ATI intermediate state. We confirmed the persistence of this intermediate state in both the bleomycin mouse model of pulmonary fibrosis and in human pulmonary fibrosis [42].

## 10. Limitations

Despite the recent progress made in studies of ATII-to-ATI differentiation, several experimental limitations must be discussed. One experimental constraint stems from the difficulty of isolating the differentiation stage in in vivo models of regeneration. As mentioned, ATII cell proliferation typically precedes ATII-to-ATI cell differentiation. In most of the studies discussed above, gene deletion was induced prior to injury, thus, any effect observed on the rate of differentiation may be confounded by an effect on the preceding proliferation phase. In other words, if gene knockout impairs both proliferation and differentiation, it may be hard to discern whether this gene plays a direct role in differentiation or whether the impaired differentiation is a consequence of impaired proliferation. Of course, this assumes that ATII-to-ATI cell differentiation requires a preceding round of replication; the extent to which ATII-to-ATI cell differentiation might occur with a preceding round of replication is unknown. 

Another limitation lies in the methods for quantification of proliferation and differentiation. Many commonly used models of lung injury are characterized by quite mild ATI cell loss and regeneration. The percent of ATI cells that are regenerated by ATII cells at the end of regeneration is approximately 3%–4% [20,21]. This is presumably the percent of ATI cells that were lost during injury (unless non-ATII cell progenitors differentiate into ATI cells). Given the low degree of ATI cell injury and regeneration, it is difficult to study the effects of interventions such as gene knockout or drugs. Highly accurate and high throughput techniques are necessary to discern a decrease in ATI cell differentiation below an already low level. In more severe injury models, the degree of ATI cell loss and regeneration may be slightly higher but is typically accompanied by loss of ATII cells, which is the cell type of interest, and this can lead to the mobilization of alternate progenitors [8,9], further confounding interpretation. A final issue relates to the quantitation of proliferation and differentiation in an unbiased manner. The above discussed studies use many different methods to quantify proliferation and differentiation. Many studies assess ATII cell proliferation by counting cell profiles per high power field or by the number of cells detectable by flow cytometry after lung digest, methods that are subject to bias [43]. Most studies quantitate ATII-to-ATI cell differentiation using images captured by fluorescent microscopy of tissue stained for a membrane ATI cell protein. The resolution of fluorescent microscopy, even confocal, is low enough that individual ATI cells cannot be counted. To overcome this limitation, many researchers measure the surface area of the labeled cells as they appear in cross section. Differentiation is then quantitated as the percent of total ATI cells that are lineage labeled. This is a reasonable approach if the denominator, the total ATI cells, remains constant, which may or not be the case in inflammatory lung injury but is not the case during compensatory regrowth after pneumonectomy in which new septa are created. Use of flow cytometry to count ATI cell number, which has been used, is likely to be quite inaccurate due to the difficulty recovering intact ATI cells after lung injury. Although counting ATII or ATI cell number [43] and measuring the actual surface area of ATI cells [21] by stereology may be the most unbiased approach available, this method requires specialized expertise and is tedious. Ideally, investigators in the field would use a common methodology that is accurate but also high throughput enough to detect small differences. In the meantime, it is important that investigators understand and recognize the biases inherent to the approaches they use.

## 11. Future Directions

In summary, based on solid studies, it appears that YAP/TAZ, Cdc42, Notch, BMP, TGFβ, and Etv5 signaling pathways are involved in the regulation of ATII-to-ATI cell differentiation during alveolar regeneration. In the future, additional research should be done to better understand this regulation. First, in most cases, the upstream stimuli that activate these pathways are unknown. In the case of the pneumonectomy model, mechanical tension is the trigger for Cdc42 and YAP activation, but it is unknown what might activate YAP/TAZ signaling in other models of lung injury. As discussed above, the signals that induce Dlk1 upregulation should ultimately be investigated and a unified picture of how these signals integrate with the other pathways involved will emerge. Kras activates Etv5 in lung cancer but it remains to be determined whether this is the critical stimulus in lung injury. In lung injury, the critical limiting step for TGFβ signaling may be at the level of activation of the ligand from its inactive form by integrins [44], but what induces that process is still unknown. The triggers for BMP and Notch signaling remain unclear. Moreover, although these key pathways have been identified, the mechanisms by which they induce ATI cell differentiation are unknown. Many of these pathways cells change in transcription, but it is unknown whether ATI cell markers are direct transcriptional targets or whether other target genes trigger additional signaling pathways that ultimately induce differentiation. Additionally, as alluded to, ATI cell differentiation entails extensive changes in both gene expression and cell morphology. The mechanisms by which the identified molecular signals regulate these changes in gene expression and cell morphology in a coordinated manner remains to be determined. 

In addition, although there is now strengthened understanding of the other individual pathway mechanisms, the sequence of these signals—whether pathways are downstream, upstream, or parallel relative to one another—is still largely unknown. As more work is done, it will become clear how these various pathways interact with each other in a coordinated manner to drive differentiation. Taken together, the work of Nan Tang et al. demonstrates that Cdc42 is upstream of YAP signaling [26,35]. YAP/TAZ signaling is known to negatively regulate Wnt/β-catenin signaling [25], and Wnt/β-catenin prevents ATI cell differentiation; thus, it is possible that one mechanism by which YAP/TAZ drives ATI cell differentiation is via β-catenin inhibition. 

It is interesting that several of the transcriptional pathways identified, β-catenin, TGFβ, and Etv5, actually inhibit differentiation and must be withdrawn to permit differentiation. Whether there is a common factor, e.g., epigenetic, that synchronizes the downregulation of these pathways, thus driving ATI cell differentiation, could be explored. Moreover, this suggests, as some have noted, that differentiation may be the default process, with active signaling required to maintain the progenitor state. This notion is supported by the concept of the ATII cell niche maintaining the ATII cell phenotype, with removal from the niche resulting in ATI cell differentiation [1,14]. Consistent with this notion is the well-established observation that ATII cells isolated from the organism differentiate into ATI cells by default in culture without specific and aggressive interventions to maintain the ATII cell phenotype [45,46,47,48].

Future studies that explore mechanisms of cell-cell crosstalk in the context of alveolar epithelial cell differentiation are warranted. In this regard, the demonstration that recruited monocyte-derived macrophages play a critical role was a seminal finding. The mediators that M2 macrophages produce, which stimulate ATII cell proliferation and/or differentiation, are yet to be determined. In addition, although this study did not find a role for T and B cells, it is highly possible that other immune cell populations are also involved. Other studies have identified prominent roles of immune cells in ATII cell proliferation [49,50]; whether they also regulate ATII-to-ATI cell differentiation should be explored. The mediators used by macrophages or other immune cells to drive ATII-to-ATI cell differentiation and whether these may be some of the known mediators discussed above should be investigated. Fibroblasts were identified to be a source of BMP during regeneration [23] and a source of Wnts during homeostasis [14,15]. The cellular source of Notch and TGFβ ligands could be studied, as could the mechanisms that trigger these cells to produce these ligands. In summary, future studies should investigate the initiation of the signaling pathways identified to regulate ATI cell differentiation, and whether cell communication is coordinated via juxtacrine, paracrine, or autocrine signals. 

Future translation of these studies into human tissues and subjects is also warranted. Ineffective alveolar regeneration is thought to underlie the pathogenesis of acute and chronic lung diseases. Once the mechanisms of normal alveolar regeneration are understood, we must begin to understand how these go awry during the pathogenesis of lung disease. We and others recently uncovered a novel transitional state occurring during ATII-to-ATI cell differentiation, with evidence to suggest that persistence of this state occurs in human pulmonary fibrosis [35,39,42]. However, additional studies to understand why this transitional state persists, how this leads to fibrosis, and whether and how the exit from this transitional state towards terminal differentiation impacts fibrosis must be performed. In addition, the ways in which the pathways now implicated in physiologic ATI cell differentiation, Notch, YAP/TAZ, etc. may become hijacked during the development of chronic lung disease should be investigated. Biorepositories of fixed human diseased lung tissue are a critical resource. Banked frozen human ATII cells can be cultured in standard two-dimensional cultures [47,51] or in three-dimensional organoids [52]. In addition, the culture of precision-cut human lung will allow examination of ATII cell behavior in its native state in the alveolus [53]. Ultimately, drugs that target the individual pathways or common convergent pathways may be used as clinical treatments to promote epithelial repair and prevent lung disease in instances of excessive lung damage.

## 12. Conclusions

**Figure 1:**
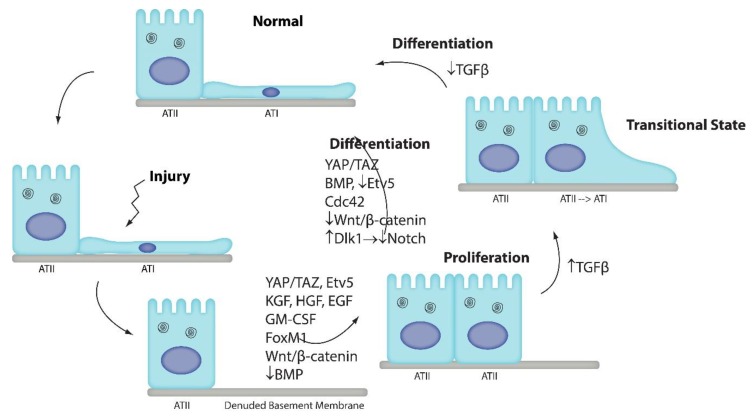
The alveolar epithelium consists of ATI and ATII cells. ATI cells are very susceptible to injury. ATII cells regenerate ATI cells. ATII cells proliferate and then differentiate into ATI cells. Many pathways have been identified that promote ATII cell proliferation: KGF [16], HGF [17,54], EGF [19,20], GM-CSF [33], FoxM1 [10], Wnt/β-catenin [13,14,15], Etv5 [37], and YAP/TAZ [24,26], whereas downregulation of BMP signaling induces proliferation [23]. Recent studies suggest that multiple pathways are also involved in ATII-to-ATI cell differentiation: YAP/TAZ [24,26], Cdc42 [35], and BMP [23] promote differentiation, whereas Wnt/β-catenin [14,15], Etv5 [37], and Notch [22] maintain the ATII cell phenotype. We recently identified a novel transitional state through which ATII cells pass as they differentiate into ATI cells [38]. Our data suggested that TGFβ promotes cell cycle exit and entry into the transitional state, whereas TGFβ downregulation permits terminal differentiation. Further studies are necessary to confirm these findings and elucidate how these pathways intersect in a coordinated manner to regulate ATII-to-ATI cell differentiation.

We know that in the lung, ATII epithelial progenitors are recruited during alveolar regeneration to replace lost ATI—and sometimes ATII—cells. Although we understand that the process of alveolar regeneration occurs in proliferation and differentiation stages, our understanding of the mechanisms of differentiation is still in its infancy. Recent studies have significantly advanced our understanding of some signaling mechanisms shown to be involved in ATII-to-ATI differentiation. Signaling pathways shown to play a role in ATII-to-ATI cell differentiation include BMP, Notch, TGFβ, β-catenin, Etv5, Cdc42, and Yap/Taz (Figure 1). Cells present in the alveolus that appear to contribute to ATI cell differentiation include fibroblasts and monocyte-derived macrophages. 

These various signaling pathways have all been shown to be involved in alveolar regeneration, with various mechanistic steps from each pathway likely interacting with one another to control differentiation. These investigations establish the foundation for further studies in this field. Using this understanding of the signaling pathways behind ATII-to-ATI differentiation during alveolar repair, similar studies can be devised to understand the interaction between these various mechanisms in coordination to control differentiation. Additionally, a strengthened understanding of these molecular pathways involved in differentiation may lead to development of new clinical treatments that accelerate lung repair in individuals with excessive lung damage.
